# Specialized microbial databases for inductive exploration of microbial genome sequences

**DOI:** 10.1186/1471-2164-6-14

**Published:** 2005-02-07

**Authors:** Gang Fang, Christine Ho, Yaowu Qiu, Virginie Cubas, Zhou Yu, Cédric Cabau, Frankie Cheung, Ivan Moszer, Antoine Danchin

**Affiliations:** 1 HKU-Pasteur Research Centre, Dexter HC Man Building, 8, Sassoon Road, Pokfulam, Hong Kong, China; 2 Plate-forme Intégration et Analyse Génomiques, Genopole, Institut Pasteur, 28 rue du Docteur Roux, 75724 Paris Cedex 15, France; 3 Unité de Génétique des Génomes Bactériens, CNRS URA2171, Institut Pasteur, 28 rue du Docteur Roux, 75724 Paris Cedex 15, France

## Abstract

**Background:**

The enormous amount of genome sequence data asks for user-oriented databases to manage sequences and annotations. Queries must include search tools permitting function identification through exploration of related objects.

**Methods:**

The GenoList package for collecting and mining microbial genome databases has been rewritten using MySQL as the database management system. Functions that were not available in MySQL, such as nested subquery, have been implemented.

**Results:**

Inductive reasoning in the study of genomes starts from "islands of knowledge", centered around genes with some known background. With this concept of "neighborhood" in mind, a modified version of the GenoList structure has been used for organizing sequence data from prokaryotic genomes of particular interest in China. GenoChore , a set of 17 specialized end-user-oriented microbial databases (including one instance of Microsporidia, *Encephalitozoon cuniculi*, a member of Eukarya) has been made publicly available. These databases allow the user to browse genome sequence and annotation data using standard queries. In addition they provide a weekly update of searches against the world-wide protein sequences data libraries, allowing one to monitor annotation updates on genes of interest. Finally, they allow users to search for patterns in DNA or protein sequences, taking into account a clustering of genes into formal operons, as well as providing extra facilities to query sequences using predefined sequence patterns.

**Conclusion:**

This growing set of specialized microbial databases organize data created by the first Chinese bacterial genome programs (ThermaList, *Thermoanaerobacter tencongensis*, LeptoList, with two different genomes of *Leptospira interrogans *and SepiList, *Staphylococcus epidermidis*) associated to related organisms for comparison.

## Background

We are facing a deluge of genome sequences. As of January 14th, 2005, the GOLD site identified 1248 completed or ongoing genome programs , and this certainly reflects only a partial view of the existing programs. While this shows that we implicitely possess an enormous wealth of information about the functions carried out by genes and genomes, the very fact that this amount is enormous makes it extremely difficult to mine that information easily. The role of specialized databases is to make this task easier for end-users. Many types of microbial genome databases exist. Most of them have been developed in a context of bioinformatics centres or laboratories purely favoring *in silico *research rather than the coupling between experiments using computers and experiments at the bench, and this is reflected in the structure and aims of the databases [1-8]. In contrast, at the onset of genome programs, we decided to set up a data structure for bacterial genomes that would help experimentalists to access knowledge on genes and genomes in an end user-oriented fashion. This was first the aim of the Colibri project, with the goal to organize *Escherichia coli *genome data, well before the whole genome sequence was known [9]. Later on, the SubtiList database was at the core of the *Bacillus subtilis *genome program data access [10]. Many databases constructed on the GenoList data schema were subsequently constructed (, [11]). However, with the exponentially growing set of genome sequences, it became important to divide up the work while maintaining the main goal of the project, that of being end-user-driven and of course, user-friendly. While an ongoing effort aims at integrating all bacterial genomes within the GenoList frame into a single database, it is important to create individual databases that could be regularly updated by a selected team of scientists (preferably those that initiated the corresponding genome program). This is particularly important for countries that are beginning to develop at a high speed into the genomics era. We took the opportunity of the creation of the HKU-Pasteur Research Centre in Hong Kong (China) to set up genome databases for the microbial sequencing projects developed in China (with databases for related organisms for comparison). Within this economic context, it was also important to take into account the cost of development. The paradigm GenoList databases are based on commercial DataBase Management Systems (DBMS) [11] and we decided to shift from a commercial DBMS to a non-commercial one, providing more freedom for the future of the project. In the present set of databases (GenoChore), emphasis is placed on retrieval of information centered on the gene as the central object, with exploration methods that query simple properties of the gene products (such as molecular mass or isoelectric point) in addition to more complex features such as the class of codon usage bias used in the gene [12]. Furthermore, queries can be made on the sequence itself using large scale analyses such as BLAST, and search for word patterns present in DNA and protein sequences.

## Construction and content

### Data schema

**Figure 1:**
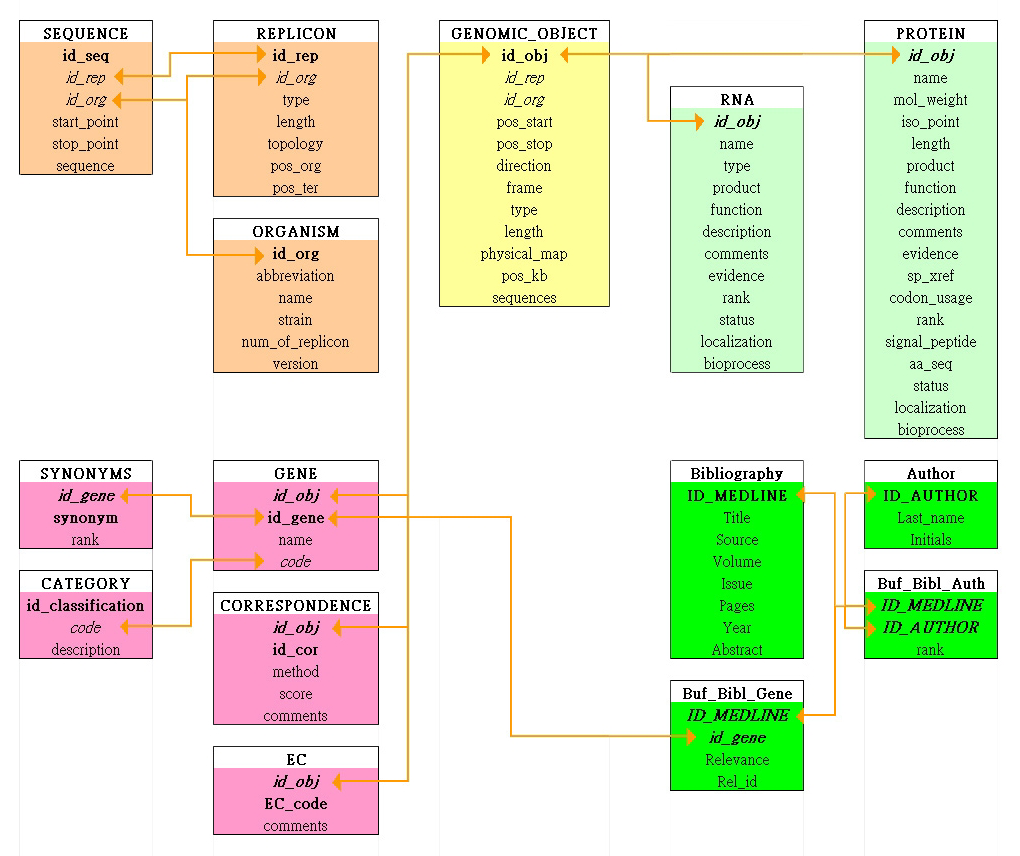
**Data Schema of the Databases. **The core object of the schema is the Genomic_object, as in GenoList. It uses pointers in the sequence that delimits several categories of objects, including protein Coding DNA Sequences (CDSs), RNAs and other objects such as transcription terminators or riboswitches.

Because we wished to shift from a commercial DBMS to an open-source one, there were some applications that could not be implemented readily due to the lack of certain advantages possessed by the commercial DBMSs. Hence, we had to alter the data structure in order to cope with this situation. The core data schema used in this work was that of GenoList version 3.1 [11], with slight modifications (Figure 1).

### Database management system

In the present GenoList databases, the DBMS used is Sybase™. While this is convenient because of excellent stability and maintenance, this may pose problems in terms of commercial policies, especially if the structure has to be exported. We therefore decided to rewrite the management of the GenoList structure using MySQL . Most function transfers were straightforward. However some functions such as nested subquery that were not available in MySQL had to be dealt with indirectly. The nested subquery has been entirely circumvented in the PERL code and is dealt with in the Extended Search algorithm by concatenating different SQL queries simply using the "AND" or "OR"connector.

### Data input

**Figure 2:**
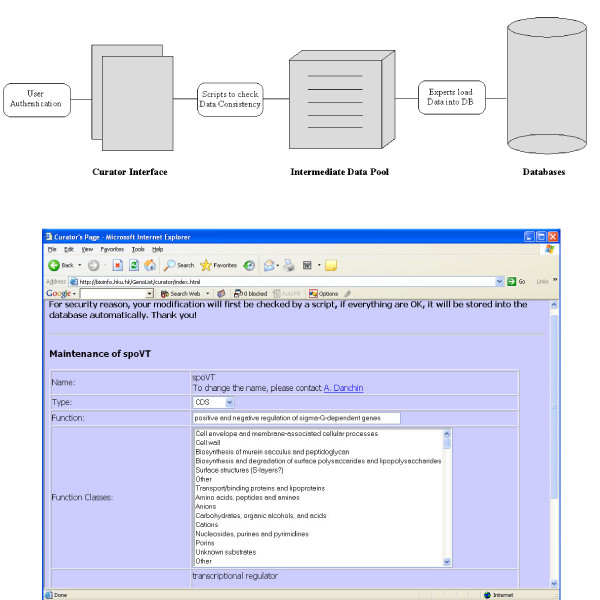
**Implementation of a Database Curator Page. **In order to help users who would participate in the improvement of the database annotation a Curator Page is provided permitting input of updated information. It is available to users after acceptation of their collaboration, through a password protected access. Once data consistency has been verified the new annotations are implemented in the current database.

Sequence and annotation data were parsed from the files extracted from the International Nucleotide Sequences Database (INSD: DDBJ/EMBL-EBI/GenBank [13,14]) with the following procedure. To get access to the INSD, the authors of a genome sequence must follow the specification of the Feature Table Definition (FTD) jointly issued by the INSD partners . The current version is Version 6.2 Oct 15, 2004. While this specification is rigid, there is still a significant degree of freedom in annotation, so that a large number of individual situations have to be taken care of semi-automatically. The basic idea of the parser is firstly to read through the input file at the INSD and check file formats. Subsequently, the information is collected and distributed into several temporary files using a set of predefined keywords and their qualifiers (i.e. those characterizing the data schema). Subsequently, a check process is initiated to identify all situations that do not fit the specifications, so that they can be corrected manually. Usually, most of the process of creating tables is automatic and only a few exceptions have to be corrected individually. A second type of input is also provided as an interactive interface to tell the database curator what information has to be collected: once collected this information can be loaded into the databases directly (Figure 2). Teraprot data are obtained from Infobiogen .

### Query methods and interface

We kept the interface of GenoList as published, except that a box providing access to protected curation of annotations is now provided, aiming initially at helping the first party (sequencing teams) annotators. The front page is made of three frames. Briefly, the vertical frame on the left contains the controls necessary to get access to the content of the database. The upper part of this frame contains text fields for querying the database according to five types of queries: gene name(s), chromosome region around a gene, chromosome region defined by positions, free text, functional classification (more detailed information about each type of query can be obtained by clicking on the question mark near the query title). The "Extended Search" button gives access to a search form allowing the user to perform multicriteria searches on all the database fields. The lower part of this frame allows one to launch sequence analysis tools: BLAST and FASTA database searches (on the sequence data), and DNA or protein pattern searches. In the former case, the user can choose to explore sequences located upstream of putative operons. In the latter case, the user can search for patterns anywhere in proteins, but also restrict the search to the beginning or end of the protein. The upper frame on the right can contain various types of information, depending on the genome and on the query. It can contain a graphical representation of a chromosome region, that can be obtained in several ways: usually from a gene in the bottom frame. This frame may contain launch forms and result lists from the sequence analysis tools available (pattern search, BLAST or FASTA scanning). The bottom frame on the right always contains detailed information about one given gene, including regularly updated BLAST searches and Teraprot reports as well as related bibliographic references. The original package managing the interface of GenoList databases was written in C/C++, following the first database schema [10], that had been adapted for use with the Sybase™ DBMS (the initial platform was using the DBMS 4^th ^Dimension™). The modification of the database schema needed for using MySQL required additional adaptations of the application interfaces. Using the original package would have required iterative work that was systematically adding complexity into the system. Current best Web interfaces and application interfaces (i.e. friendly for sharing parties) are often based on Perl scripts. For this reason a new core management script was recreated, written in Perl, while keeping the package architecture and the Web interfaces. Among other languages that have comparable functionalities, the choice of Perl to create the system was motivated by its powerful capability to glue different programs or scripts together. In addition it is widely used by the INSD, and at the European Bioinformatics Institute in particular within the BioSapiens program . Furthermore, this choice allowed us to keep the optimized fast C code that has been constructed for searching pattern (strings of symbols) inside the DNA or amino acids sequences. The GenoList C/C++ package chose to use the GD library  for generating graphic representations of genome regions. The GD graphics library is an open source library which allows programmers to easily generate PNG, JPEG, and WBMP images from many different programming languages. We used here a newer version of the same library (perl module perl-GD version 2.11) to make use of its improvements in creating dynamic pictures. In rewriting the core of the program we used the Perl module DBI . A DBI is a middle layer between the outside applications and the communicator (DBD). Different DBMSs have their own communication mechanism to talk with outside applications, and in the present version the choice of the DBI module has been implemented in such a way that we could change the DBMS if necessary with minimal work. In this way, when changing the DBMS, it will only be necessary to tell the DBI about the specifications of the new DBMS without having to modify any other code. Finally, we used the Perl module CGI to facilitate the production of the WebPage interfaces. As a consequence further developments of the GenoChore package should be performed with minimal effort.

## Utility and discussion

### Data schema

In the original GenoList structure, the central table corresponding to genomic objects carried all relevant features that are associated to genes and gene products. For the sake of future developments and to accommodate new feature annotation present in genome flatfiles, we separated this table into several gene product tables, specific for RNAs and proteins. The current data structure remains open to include tables for other types of data, such as regulation properties annotations when they will become available. Figure 1 displays a diagram of the current generic database schema (we did not show tables that remain empty for want of annotation data). As expected for a database meant to provide knowledge from genome programs, the central tables are focussing on genomic objects, the main one corresponding to protein Coding DNA Sequences (CDSs). To match this structure, the information present in the flat files created by the sequencing consortia, and present in the INSD, is split into three parts, namely, a) genomic objects, i.e. what we see in a chromosome, at precisely identified positions in the genome sequence (depending on the annotation tools available to the consortia), such as a CDS, a promoter, a terminator, a tRNA, an sRNA etc.; b) genome annotations, i.e. protein, RNA and other bio-molecules' products, functions, comments and so on; c) relations between genomic objects: e.g. the typical concept of gene requires its association to a promoter, a terminator and usually a CDS. In this representation, a set of genome objects' identifiers (ids) is used to represent a gene. This facilitates the association of genomic objects together with much more sophisticated relationships into more complex structures, when required.

It is important here to notice that, in contrast to a rather ubiquitous practice, we explicitely separate between Open Reading Frames (ORFs) that are simply sequences multiple of 3 between two termination codons (TAA, TAG and TGA) and CDSs, that begin with a specific codon, usually ATG (in the DNA text), preceded by a ribosome binding site (RBS), typically AAGGAGGT in many bacterial genomes. One must remember that in most genomes the beginning of CDSs has not been experimentally identified. Identification of CDS starts is however much easier in low G+C Firmicutes that do not possess a counterpart of ribosomal protein S1 found in gamma proteobacteria [15]. In the same way, G+C-rich organisms have usually long ORFs, but the CDSs they harbour are usually highly enriched in A+T at the third codon position. Some caution, therefore, should be exerted by users when using the information collected in the databases about the beginning of proteins in these organisms (for example in the *Streptomyces coelicolor *database, CoeliList).

### Nomenclature: naming genes

Users know that the system used for naming genes in genome databases is extremely unwieldy and completely lacks standardization. This is usually because genes are simply labelled in databases by access numbers corresponding to the annotation phase of the relevant genome program (e.g. *PA3004 *for a gene found in the genome of *Pseudomonas aeruginosa*). In the absence of knowledge of a gene name it takes some time to identify it (often using BlastP), for example when aiming at the study of its neighborhood ((i.e. proximity of an object or a relationship with others sharing the same conceptual space, including presence in a common article [12]). Naturally, because most genes have never been experimentally identified in the majority of the newly sequenced genomes, this approach is certainly safer than giving a name without proper identification criteria. However it is extremely useful for scientists studying a genome to start from "islands of knowledge", with genes with a known background, reflected by a known gene (and a gene name has usually been coined by experimentalists for that gene). For this reason, we decided to use a conservative approach, using bidirectional best Blast hits of the genome of interest with model genome (*Escherichia coli *K12 and *Bacillus subtilis *168). Orthologues were identified as reciprocal best hits [16] (using a global alignment where the gaps on the edges of the largest sequence are ignored) with at least 50% identity in amino acid sequence and less than 20% difference in protein length. When possible, in order to increase the likelihood of the putative identification we used a second well known representative of the genome under study and looked for orthologues between every pair of each of the two triplets (i.e. between each pair of the three organisms: the organism of which the database is constructed, *B. subtilis *for Firmicutes and another organism of the same family, such as *Listeria monocytogenes*, and *E. coli *for gamma-proteobacteria, with another one of the same family, such as *Photorhabdus luminescens*). Finding putative orthologues in the three organisms was considered as substantiating evidence for the use of a gene name. Then, in each triplet, we did not transfer the model organism gene name to all orthologues that were not simultaneously present in the three genomes or that gave different correspondences in different comparisons. In another comparison where the orthologues were found with at least 50% similarity, the model organism gene names to be transferred were preceded by the letter '*y*'. In order to help users recognize gene names (and all the knowledge they associate with those names) we used as reference names those in the model bacteria, trying to comply as much as possible with the names used at SwissProt in the HAMAP project [17].

This allows the users to have "anchor" points to start to use the databases in a more efficient way. Naturally, the names previously used in the corresponding genome programs are kept as synonyms, so that access to the sequences with these names is still allowed. For example, in AeruList, gene *rpsA *can be accessed directly or using its synonym *PA3162*: it is then found downstream of *cmk *(a context similar to that found in many Gram negative bacteria) and upstream of *himD*. We are aware that some erroneous identification (or propagation of erroneous identifications) must have occurred in some cases, but we think that this is a trade-off (which will be continuously corrected) for a much more user-friendly usage of the databases. A '*y*' letter starting a gene name indicates that it has not been experimentally identified, nor convincingly identified after *in silico *analysis yet. We provide curation pages (see below) to help users to correct annotation errors and improve annotation in a continuous way.

### Functional categories and bioprocesses

An important feature for allowing users to explore biological functions is to investigate the genes neighborhoods [12]. Related functions are often coded by genes in close vicinity in the chromosome. We therefore used the GenoList table for functional categories, that allows the user to make links with the roles of proteins in the cell. The functional classification used in some of the present databases has been created by superimposing the functional classification (ontology) created for SubtiList, and that of *Escherichia coli *created by Monica Riley and her collaborators [18] (Additional file). In addition we created a field for the ontology describing underlying bioprocesses: explore, sense, shape, circulate, excrete, replicate, grow, respire, manage energy, store, scavenge, maintain, protect, control. They will be used in the future to color the arrows indicating genes in the picture of the region surrounding a gene of interest, allowing the user, at a glance, to have a rough idea of the processes encoded in the corresponding region.

### Queries using mining algorithms

In addition to using keyword queries or sequence tags (such as molecular mass or isoelectric point of a protein) the database provides a versatile way to identify sequences from the biological knowledge viewpoint. In particular, as in many other databases, it allows the user to use Fasta, BlastP and BlastN to compare a sequence of interest to that of those present in the database. Furthermore, in contrast to most cases, it allows the user to extract information using motifs, that can be either continuous or discontinuous (e.g. finding all proteins with motif CXXCHX_12–25_C). This facility has already, in a quite unobtrusive but efficient way, permitted discovery of many unexpected functions. We have also provided means to explore the beginning and the end of protein sequences, as well as DNA regions upstream of putative operons, computed as strings of genes transcribed in the same orientation and separated by a maximum number of nucleotides (60 nt by default).

### Automatic updates

Genome annotation is continuously updated by scientists all over the world, at a time when new genome sequences appear every three days or so. In order to cope with this enormous flux of information, a facility for browsing automatically new entries in major data libraries has been implemented. In the gene information panel, where each gene of interest is described after being identified as the result of a query (including resulting from a Blast or Pattern search), an "Automatic Blast" link provides a list of updated blast searches against the UniProt library (SWISSPROT+TREMBL). In addition, when the genome belongs to the 'Teraprot' Smith and Waterman Z-score family , the corresponding links (that are statistically much more significant than the results of Blast searches) are provided, allowing the user to look for remote kinships.

To discuss the use of the databases we shall restrict our exploration to two databases from the package. LeptoList, that comprises two genomes (each one having two chromosomes) for Bacteria, and CunicuList, that describes the genome sequence and annotation of a small eukaryote.

### An example: LeptoList

LeptoList is the reference database dedicated to the genome of *Leptospira interrogans *serovar Lai, the paradigm of leptospirosis causative agents [19]. It is presented together with a second sequence, that of *L. interrogans *serovar Copenhageni in order to allow easy comparison [20]. The WWW interface takes into account the fact that *L. interrogans *has two chromosomes (this feature was not yet displayed in GenoList databases). Using the regular comparison to the CDS to the non-redundant INSD protein database allowed us to suspect that a significant proportion of the short putative CDSs in the genome are likely ORFs and not authentic CDSs. This fits with the recent sequencing of the second Leptospira genome [20,21].

A couple of examples of its use are given here. We looked for counterparts of RRF, the ribosome release factor. In order to find the gene we used a known sequence, from *B. subtilis *(*frr *gene product) and compared it using BLAST with the functionality implemented in LeptoList. This search led to a single gene, LA3295, located downstream of gene *pyrH *(as in most other bacterial genomes). This synteny is obviously highly significant. In the same way, the gene immediately upstream from *pyrH *(LA3297), as in other bacteria, is likely to be coding for elongation factor EFTs (*tsf*). When curating the database, we suggest to the curator that it would be of excellent policy to replace the gene numbers by the corresponding gene name. In another type of investigation, looking for patterns of the type TTGACA (1 ambiguity) – 17 nt – TATAAT# (1 ambiguity) (consensus sequence of the σ^70^-type promoter) in the 300 nt region upstream of genes revealed 70 sequences in chromosome I, many of which are likely to be promoters (at least they would be good guesses to start investigating promoters in *L. interrogans*). In the same way, the putative DNA binding site located in the 300 nt nucleotide region upstream of genes, TGTGA (1 ambiguity) – 2 nt – KK – 2 nt – TCACA (1 ambiguity) (consensus sequence of the CAP/FNR family of transcriptional regulators), yielded 130 matches in chromosome I of serovar Lai and 72 matches in serovar Copenhageni and 2 in chromosome II of serovar Lai and 0 in serovar Copenhageni, allowing one to start investigating possible regulatory elements.

This result is interesting as it suggests that chromosome I genes are submitted to a regulation recognizing that particular DNA-protein binding site. Furthermore, most genes found with the site in serovar Copenhageni are also found in serovar Lai, with sometimes several repeats in the latter, occuring upstream of some genes (such as *fadH *or *prfC*), accounting for the higher total number of putative binding sites in that organism. It seems most interesting that genes involved in the control of respiration (cytochrome c biosynthesis), control of the TCA cycle (pyruvate dehydrogenase synthesis), control of the coupling between translation and transcription (stringent control) or translation itself (release factor 3 synthesis) are present in the list. While there are several putative adenylyl cyclase genes present in the organism, as well as several homologs of *crp*, it is plausible to propose that cAMP plays an important role in the life cycle of *L. interrogans*, perhaps suggesting ways to allow multiplication on plates of this elusive organism.

LeptoList is accessible at the URL 

### CunicuList: a database for a small eukaryote genome

The GenoList structure has been initially constructed for organizing sequence data from prokaryotic genomes. However it may be extended to other organisms as well (the "genomic object" type must be extended accordingly). We have therefore tested the implementation of the structure for the genome of *Encephalitozoon cuniculi*, belonging to the Microsporidia taxon. Eleven chromosomes are present in this organism. Extraction of information is similar to that from other databases. For example we looked for counterparts of genes involved in tRNA modification (often essential genes). Using MesJ (TilS) [22] as well as TrmU [23] we found that gene Ecu03_1240 is most probably involved in driving the codon and amino acid specificity of a tRNA (possibly isoleucine or lysine tRNA). In the same way we could predict that gene Ecu07_1610 codes for synthesis of dihydrouridine in tRNA, a general feature of tRNA structure, because of its similarity with the *yacF B. subtilis *gene. Looking for counterparts of genes in the methionine salvage pathway [24], we failed to identify any gene that would code for the enzymes of the pathway, indicating that the parasite obtains all the metabolites derived from S-adenosylmethionine from its host. This is substantiated by the fact that the genes needed to synthesize queuosine [25] are apparently absent from the genome. Some organisms do not use this major tRNA modification, but this could be an interesting information for identification of drug targets against the parasite, since this suggests that those metabolites have to be transported into the cell by specific permeases.

### Database curation

Several other similar bacterial databases are accessible at URL . Table 1 presents the list of microbial databases that are available at the Bioinfo server of the University of Hong Kong.

**Table 1:** List of databases present at the Bioinfo server The GenoChore suite presented here manage bacterial genome data, except for CunicuList, which presents the sequence and annotation data of the small eukaryote *Encephalitozoon cuniculi*.

**AeruList**	*Pseudomonas aeruginosa *PA01	EMBL:AE004091
**AnthraList**	*Bacillus anthracis *str. Ames	EMBL:AE016879
**CampyloList**	*Campylobacter jejuni *NCTC 11168	EMBL:AL111168
**CereList**	*Bacillus cereus *ATCC 14579	EMBL:AE016877
**CholeList**	*Vibrio cholerae*	EMBL:AE003852 , EMBL:AE003853
**CoeliList**	*Streptomyces coelicolor *A3(2)	EMBL:AL645882
**DiphteList**	*Corynebacterium diphtheriae *NCTC 13129	EMBL:BX248353
**CunicuList**	*Encephalitozoon cuniculi*	EMBL:AL391737 , EMBL:AL590442 , EMBL:AL590443 , EMBL:AL590444 , EMBL:AL590445 , EMBL:AL590446 , EMBL:AL590447 , EMBL:AL590448 , EMBL:AL590449 , EMBL:AL590450 , EMBL:AL590451
**InfluList**	*Haemophilus influenzae *Rd KW20	EMBL:L42023
**LeptoList**	*Leptospira interrogans *Lai str. 56601	EMBL:AE010300 , EMBL:AE010301
	*Leptospira interrogans *Fiocruz L1-130	EMBL:AE016823 , EMBL:AE016824
**MeningoList**	*Neisseria meningitidis *MC58	EMBL:AE002098
**PutidaList**	*Pseudomonas putida *KT2440	EMBL:AE015451
**SepiList**	*Staphylococcus epidermidis *ATCC 12228	EMBL:AE015929
**SubtiList**	*Bacillus subtilis *str. 168	EMBL:AL009126
**ThermaList**	*Thermoanaerobacter tencongensis *MB4	EMBL:AE008691
**VulnifiList**	*Vibrio vulnificus *YJ016	EMBL:BA000037 , EMBL:BA000038
**XylelList**	Xylella fastidiosa 9a5c	EMBL:AE003849

Despite of – or because of – the large number of genome programs, once a sequence has been deposited at the INSD, its annotation is seldom updated. This is because the cost of curating annotations is extremely high, and usually not considered, despite its enormous importance. One of our aims was therefore to allow curation by selected teams by creating a curator page where such teams would input their annotations, that would then be propagated to the databases. The basic schema of the curator interface is shown in Figure 2. In order to preserve the quality of the input data, potential users are asked to write to the database's webmaster to ask for account and passwords. We kept the interface of GenoList as published, except that a box providing access to protected curation of annotations is now provided, aiming initially at helping the first party (sequencing teams) annotators. If this works to our satisfaction this will be extended to selected third party annotators. Subsequently, on a yearly basis (or more frequently if needed) the collected re-annotation of the curators would be submitted as a new version of the same genome to the INSD. We hope that this service will be useful for the scientific community as a whole.

## Conclusions

A set of 17 specialized end-user-oriented microbial databases (including one instance of Microsporidia) has been implemented in Hong Kong. They allow one to browse genome sequence and annotation data using the most frequent queries that end-users would like to ask. In addition they provide a weekly update of searches against the world-wide protein sequences data libraries, allowing one to monitor annotation on genes of interest. Finally, they allow users to search for patterns in DNA or protein sequences present in the databases. All comments, bug reports and suggestions for improvement are more than welcome: this work is meant to be useful for the community of microbiologists interested in genomics.

## Competing interests

The author(s) declare that they have no competing interests.

## Authors' contributions

GF wrote the parsers used to create the preliminary C/C++ MySQL package, and created important sections of the Perl package; CH created the procedure for renaming orthologs with reference to accepted names for model bacteria (*Bacillus subtilis *and *Escherichia coli*), created the link to Teraprot for identification of gene functions, and implemented parts of the PERL package; YQ implemented parts of the Perl package; CC and VC implemented most of the databases into the core structure; ZY wrote part of the new parsers, implemented the two chromosomes of LeptoList by changing the data structure in the database and set up with CC the first LeptoList database; FC set up and administered the Apache web server and MySQL database; IM over the years designed most of the GenoList data schema and user interface; AD was at the origin of the project, participated in the design and evolution of the data schema, was the systematic tester and end-user and wrote the core of the article.

## Supplementary Material

Additional File 1 **Functional categories. **The genes' roles are listed into six major categories. The three first ones are directly linked to biological roles, while the remaining categories are created *ad hoc*: adaptation to atypical conditions correspond to miscellaneous roles, while the two last categories correspond to roles that have not yet been ascribed to genes because of lack of *in vivo *or *in silico *data Click here for file
