# Supplementary material for: Specialized microbial databases for inductive exploration of microbial genome sequences
Source: BMC Genomics. 2005 Feb 7;6:14. doi: 10.1186/1471-2164-6-14 (PMC549560; doi:10.1186/1471-2164-6-14)
Supplement: Additional File 1 — Functional categories. The genes' roles are listed into six major categories. The three first ones are directly linked to biological roles, while the remaining categories are created ad hoc: adaptation to atypical conditions correspond to miscellaneous roles, while the two last categories correspond to roles that have not yet been ascribed to genes because of lack of in vivo or in silico data [file 1471-2164-6-14-S1.doc]

| 1. **Cell envelope and membrane-associated cellular processes**  1.1 *Cell wall*  1.1.1 Biosynthesis of murein sacculus and peptidoglycan  1.1.2 Biosynthesis and degradation of surface polysaccarides and  lipopolysaccharides  1.1.3 Surface structures  1.1.4 Other  1.2 *Transport/binding proteins and lipoproteins*  1.2.1 Amino acids, peptides and amines  1.2.2 Anions  1.2.3 Carbohydrates, organic alcohols, and acids  1.2.4 Cations  1.2.5 Nucleosides, purines and pyrimidines  1.2.6 Porins  1.2.7 Unknown substrates  1.2.8 Other  1.3 *Sensors (signal transduction)*  1.3.1 Sensors for chemicals  1.3.2 Mechanosensors  1.3.3 Electrical sensors  1.3.4 Other  1.4 *Membrane bioenergetics (electron transport chain and ATP synthase)*  1.4.1 ATP-proton motive force interconversion  1.4.2 Electron transport  1.4.3 Photosynthesis  1.5 *Motility and chemotaxis*  1.5.1 Chemotaxis  1.5.2 Aerotaxis  1.5.3 Motility  1.5.4 Swarming  1.6 *Protein secretion*  1.6.1 Protein and peptide secretion and trafficking  1.6.2 Toxin production and resistance  1.7 *Cell division*  1.7.1 Cytoskeleton  1.7.2 Cell division  1.8 *Horizontal gene transfer*  1.8.1 Competence  1.8.2 Conjugation  1.8.3 Phage / plasmid invasion and immunity  1.9 *Collective behaviour*  1.9.1 Biofilm formation  1.9.2 Ring formation and extracellular matrix   - 1. *Differentiation*      1. Isolated sporulation      2. Aggregation and sporulation | 2. **Intermediary metabolism**  2.1 *Metabolism of carbohydrates and related molecules*  2.1.1 Main glycolytic pathways  2.1.1.1 Glycolysis/gluconeogenesis  2.1.1.2 Pentose-phosphate pathway  2.1.1.3 Entner-Doudoroff pathway  2.1.1.4 Fermentation  2.1.1.5 Pyruvate dehydrogenase and input/output into the TCA cycle  2.1.2 TCA and related cycles  2.1.2.1 TCA cycle  2.1.2.2 Glyoxylate cycle  2.1.2.3 Other  2.1.3 Metabolism of carbohydrates and related compounds  2.1.3.1 Carbohydrates (other than glucose)  2.1.3.2 Sugar alcohols  2.1.3.3 Sugar acids  2.1.3.4 Amino sugars  2.1.3.5 Biosynthesis and degradation of polysaccharides  2.1.3.6 Other  2.2 *Metabolism of amino acids and related molecules*  2.2.1 Amino acid biosynthesis and salvage  2.2.1.1 Aromatic amino acid family  2.2.1.2 Aspartate family  2.2.1.3 Glutamate family  2.2.1.4 Pyruvate family  2.2.1.5 Serine family  2.2.1.6 Histidine family  2.2.1.7 Other  2.2.2 Polyamines biosynthesis  2.2.3 Degradation of aminoacids and related molecules  2.2.3.1 Degradation of proteins, peptides, and glycopeptides  2.2.3.2 Degradation of aminoacids  2.2.3.3 Degradation of polyamines  2.3 *Metabolism of nucleotides and nucleic acids*  2.3.1 Biosynthesis and salvage of purines, pyrimidines,  nucleosides, and nucleotides  2.3.1.1 Purine ribonucleotide biosynthesis  2.3.1.2 Pyrimidine ribonucleotide biosynthesis  2.3.1.3 Deoxyribonucleotides biosynthesism  2.3.1.4 Salvage of nucleosides and nucleotides  2.3.2 Degradation of purine and pyrimidines  2.3.3 Sugar-nucleotide biosynthesis and conversions  2.3.4 Other  2.4 *Metabolism of lipids*  2.4.1 Fatty acid and phospholipid metabolism  2.4.1.1 Biosynthesis  2.4.1.2 Degradation  2.4.2 Terpenes and related-molecules  2.4.3 Other  2.5 *Metabolism of coenzymes and prosthetic groups*  2.5.1 Biotin  2.5.2 Folic acid  2.5.3 Lipoate  2.5.4 Menaquinone and ubiquinone  2.5.5 Molybdopterin  2.5.6 Pantothenate and coenzyme A  2.5.7 Pyridoxine and derivatives  2.5.8 Riboflavin, FMN, and FAD  2.5.9 Glutathione  2.5.10 Thiamine  2.5.11 Pyridine nucleotides  2.5.12 Heme, porphyrin, and cobalamin  2.5.13 Chlorophyll  2.5.14 Other  2.6 *Specific pathways*  2.6.1 Nitrogen fixation  2.6.2 Methanogenesis  2.6.3 Chemoautotrophy  2.6.4 Other  2.7 *Metabolism of phosphate*  2.8 *Metabolism of sulfur* | 3. **Information transfer pathways**  3.1 DNA metabolism  3.1.1 DNA replication  3.1.2 DNA recombination  3.1.3 DNA packaging and segregation  3.1.4 DNA proof-reading and repair  3.1.5 DNA restriction/modification  3.1.6 Degradation of DNA  3.2 RNA metabolism  3.2.1 Transcription  3.2.1.1 Regulation  3.2.1.1.1 Regulatory proteins (DNA interaction)  3.2.1.1.2 Regulatory proteins (RNA interaction)  3.2.1.1.3 Regulatory RNAs  3.2.1.2 Initiation  3.2.1.2.1 Sigma factors  3.2.1.2.2 Other  3.2.1.3 Elongation  3.2.1.3.1 RNA polymerase (core enzyme)  3.2.1.3.2 Transcription / translation coupling  3.2.1.3.3 Other  3.2.1.4 Termination  3.2.2 Processing and degradation of RNA  3.2.2.1 RNA chaperones  3.2.2.2 Ribonucleases  3.2.2.3 Other  3.2.3 RNA modification  3.2.3.1 tRNA modifications  3.2.3.2 rRNA modifications  3.2.3.3 Other modifications  3.3 Protein synthesis, maturation and folding  3.3.1 Ribosomal proteins  3.3.2 Aminoacyl-tRNA synthetases  3.3.3 Initiation  3.3.4 Elongation  3.3.5 Termination  3.3.6 Protein modifications  3.3.6.1 Maturation  3.3.6.2 Chemical modifications  3.3.7 Protein folding  3.3.8 Protein repair | 4. **Other functions**  4.1 Adaptation to atypical conditions  4.2 Detoxification  4.3 Antibiotic production  4.4 Phage-related functions  4.5 Transposons and ISs  4.6 Miscellaneous  4.6.1 Plasmid-related functions  4.6.2 Pathogenesis | 5. **Similar to unknown proteins**  5.1 From the same organism  5.2 From other organisms | 6. **No similarity**  6.1 Unknown protein confirmed by proteomics  6.2 Hypothetical (Doubtful CDS) |
| --- | --- | --- | --- | --- | --- |
